# Incidence of injury and illness during the 2013 world dwarf games

**DOI:** 10.1186/s40621-019-0191-1

**Published:** 2019-04-02

**Authors:** Mathew R. Saffarian, Jensen J. Swampillai, Michael T. Andary, Jim R. Sylvain, Salina E. Halliday, Brian Bratta

**Affiliations:** 1 4660 S. Hagadorn Rd. St. 520, East Lansing, MI 48823 USA; 2 2001 Northamption Way, Lansing, MI 48912 USA; 3 20 Alumni Arena, University at Buffalo, Buffalo, NY 14260 USA

**Keywords:** Skeletal dysplasia, World dwarf games, Athletics, Exercise, Sports, Dwarfism, Achondroplasia

## Abstract

**Background:**

Dwarfism, or skeletal dysplasia, is a term used to describe short stature. Injuries to athletes with disabilities and medical co-morbidities, such as those present in the dwarf population, can have significant consequences on functionality. The main objectives of this retrospective descriptive study were to 1) evaluate the safety of athletic participation among athletes with skeletal dysplasia, 2) investigate the incidence and characteristics of injuries and illnesses among athletes with skeletal dysplasia during the 2013 World Dwarf Games held on the campus of Michigan State University, 3) describe details and overview of the World Dwarf Games, and 4) identify possible safety and rule issues to improve safety at future World Dwarf Games.

**Methods:**

This was a retrospective review of case series interactions between dwarf athletes and the medical staff present at the 2013 World Dwarf games from August 3–10, 2013. Injury incidence rates were calculated by dividing the number of incident injuries by total athlete-competitions. Epidemiologic incidence proportion calculations were used to measure average injury risks.

**Results:**

A total of 24 competition related injuries were recorded among the 409 athletes. Only 1 illness (otitis media) was reported during the week of games. The overall injury incidence rate was found to be 0.78 injuries per 100 athlete-competitions. The overall epidemiologic incidence proportion was 5.9% (7.2% for males, 3.0% for females). The most common type of injury was a muscle/tendon strain (41.7% of all injuries). The sport with the most reported injuries was soccer with 4.63 injuries per 100 athlete-competitions.

**Conclusions:**

Based on the data collected, it does appear that athletes with skeletal dysplasia can safely participate in the events offered during the World Dwarf Games. None of the reported injuries or illnesses precluded the athletes from returning to play. Data collected at future competitions will help identify trends, which may lead to rule changes to improve safety and a decrease in injuries. Adding a designated spectator area for athletes as well as modifying rules to prevent excessive physical contact in soccer and basketball competitions may reduce the incidence of injury.

## Background

Dwarfism, or Skeletal Dysplasia (SD), is a term used to describe short stature, which is defined as three or more standard deviations below the mean height for age (Emedicine, 2015). There are multiple etiologies of SD, including metabolic or endocrine disorders, birth defects, and chromosomal disorders (Emedicine, 2015). Over 400 types of SD have been identified (Emedicine, 2015).

The incidence of SD is approximately 1 in every 4000 to 5000 births (Emedicine, 2015). Many cases are not identified until childhood. The four most common types of SD are achondroplasia, osteogenesis imperfecta, achondrogenesis, and thanatophoric dysplasia (Emedicine, 2015). Achondrogenesis and thanatophoric dysplasia are lethal forms of SD, while achondroplasia is the most common type of non-lethal SD (Emedicine, 2015).

Due to skeletal abnormalities, people with SD can have functional abnormalities and medical co-morbidities. Clinical complications of SD are numerous and include enlarged cranium with hydrocephalus, genu varum, hearing impairments, cataracts, atlantoaxial instability, exaggerated cervical and thoracolumbar kyphosis, endocrine disorders, metabolic disorders, and cognitive impairments (Emedicine, 2015).

Orthopedic and neurological abnormalities can be functionally limiting in the SD population (Hunter et al., 1998). Cervicomedullary compression and spinal stenosis can be seen at an early age in the achondroplasia population (Hunter et al., 1998). It has been estimated that by the age of 12, approximately 20% of children with achondroplasia will begin to experience symptoms of spinal stenosis (Hunter et al., 1998). This number rises up to 50% by adulthood (Hunter et al., 1998). For multiple reasons obesity has become prevalent among those affected with SD (Pauli, 1998).

The Little People of America (LPA) and the Dwarf Athletic Association of America (DAAA) have teamed together to develop, promote, and provide quality amateur level athletic competition opportunities for dwarf athletes in the United States in an effort to prevent obesity and increase physical fitness within the SD population. Competitions include the National Dwarf Games, which are held annually in the United States, and the World Dwarf Games, which are held every four years with worldwide participation among dwarf athletes.

The incidence of injuries and illness among Paralympic Game athletes with physical impairments and disabilities has been well documented (Webborn et al., 2012; Derman et al., 2013; Willick et al., 2013). This has lead to improvement among athlete preparation and safety during the Paralympic Games. For example, in 2005, rule changes and improved protective equipment in ice sledge hockey resulted in remarkable reductions in lower limb injuries in successive Paralympic Winter Games (Willick et al., 2013; Webborn & Van de Vliet, 2012).

Previous studies have compared the inherent risk of participation in athletic events among athletes with disabilities to “able bodied” athletes and shown the risk of injury to be almost equivalent (Webborn et al., 2006). However, injuries to athletes with disabilities and substantial medical co-morbidities, such as those present in the dwarf population, may have significant consequences on functionality.

How athletic competition has affected those with SD has yet to be investigated. The main objectives of this retrospective descriptive study were to 1) evaluate the safety of athletic participation among athletes with skeletal dysplasia, 2) investigate the incidence and characteristics of injuries and illnesses among athletes with skeletal dysplasia during the 2013 World Dwarf Games held on the campus of Michigan State University, 3) describe details and overview of the World Dwarf Games, and 4) identify possible safety and rule issues to improve safety at future World Dwarf Games.

## Methods

### Study design

This study was a retrospective review of case series interactions between dwarf athletes and the medical staff present at the 2013 World Dwarf Games from August 3–10, 2013. Injury incidence rate was calculated as the number of incident injuries divided by total athlete-competitions. An athlete-competition was defined as one athlete participating in one competition. Certain sports, such as swimming and track & field, had multiple competitions. Epidemiologic incidence proportion was used as a measure of risk. It was calculated by dividing the number of athletes injured by the number of athletes at risk during the 2013 World Dwarf Games. Reported injuries that occurred outside of competition were noted, but not included when calculating injury incidence rates and epidemiologic incidence proportions.

### Study participants

Prior to the start of the 2013 World Dwarf Games, the DAAA approached the Medical Director of the Games requesting that interactions between the athletes and the medical staff be documented as part of a quality improvement project. An attempt was made to get approval from the Michigan State University Institutional Review Board. However, due to a lack of resources, language barriers, and an inability to have face-to-face interactions with all athletes prior to the games to get written informed consent, we decided not to prospectively collect data. Approximately one year following the completion of the Games, it was decided to retrospectively analyze the medical staff-athlete interactions. Approval was obtained from the Michigan State University Institutional Review Board. The Michigan State University Department of Physical Medicine & Rehabilitation, along with the help of the Michigan State University Athletic Staff, conducted this study.

### Sporting events and classification

Sporting events at the 2013 World Dwarf Games included archery, badminton, basketball, boccia, floor hockey, kurling, powerlifting, shooting, soccer, swimming, table tennis, track and field, and volleyball. Events were modified to accommodate the impairments present among the SD population. Athletes were classified into age specific groups: Futures (6 and younger), Junior A (7–11 years old), Junior B (12–15 years old), Open (any age), and Masters (35 and older). Participants in each sport per division are shown in Table 1. A classification system was used for badminton, table tennis, boccia, swimming, and track and field in the Masters and Open divisions.

**Table 1:** The number of athlete-competitions during the 2013 World Dwarf Games in each sport and overall. While there were 409 total athletes that participated, many of the athletes competed in multiple events

	Futures	Junior A^a^	Junior B^a^	Open	Masters	Total # of Athlete-Competitions
Archery	0	0	21	54	49	124
Badminton	0	22	50	219	36	327
Basketball	0	27	42	133	0	202
Boccia	10	24	49	117	61	261
Floor Hockey	3	23	29	128	0	183
Kurling	7	10	14	0	0	31
Powerlifting	0	0	10	29	7	46
Shooting	0	0	10	33	23	66
Soccer	6	37	42	174	0	259
Swimming	6	111	82	251	23	473
Table Tennis	0	11	25	68	14	118
Track and Field	31	192	196	353	70	842
Volleyball	0	15	24	121	0	160
Total						3092

^a^There was an additional age division “Juniors – ages 10–12” for swimming as well as track & field events. Participants from these events were added to Junior A if ages 10–11 or Junior B if age 12

**Fig. 1:**
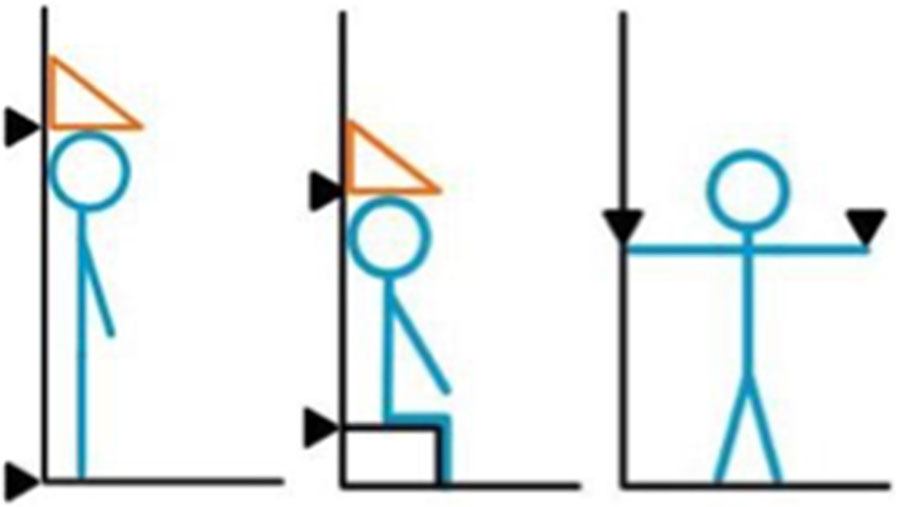
Upper and lower body classifications for Open and Masters division athletes. Measurements of standing height, sitting height, and arm span were used to determine lower and upper body classifications for Open and Masters division athletes

Lower body classification was determined by leg length (difference between standing height and sitting height) and was used for Open and Masters division athletes in badminton, table tennis, and track. Upper body classification was determined by arm span (fingertip to fingertip with arms abducted 90 degrees) and was used for Open and Masters division athletes in boccia, field competitions, and swimming (Fig. 1). Three classes were used for the upper body and lower body classification system (Table 2).

**Table 2:** Upper and lower body classification systems used for badminton, table tennis, boccia, swimming, and track and field in the Masters and Open divisions

	Arm Span - Males	Arm Span - Females
Class 1	Up to 106.0 cm	Up to 97.0 cm
Class 2	106.1 cm – 130.0 cm	97.1 cm – 120.0 cm
Class 3	130.1 cm and above	120.1 cm and above
	Leg Length - Males	Leg Length - Females
Class 1	Up to 41.0 cm	Up to 39.0 cm
Class 2	41.1 cm – 53.0 cm	39.1 cm – 49.5 cm
Class 3	53.1 cm and above	49.6 cm and above

### Data sources and variables

**Fig. 2:**
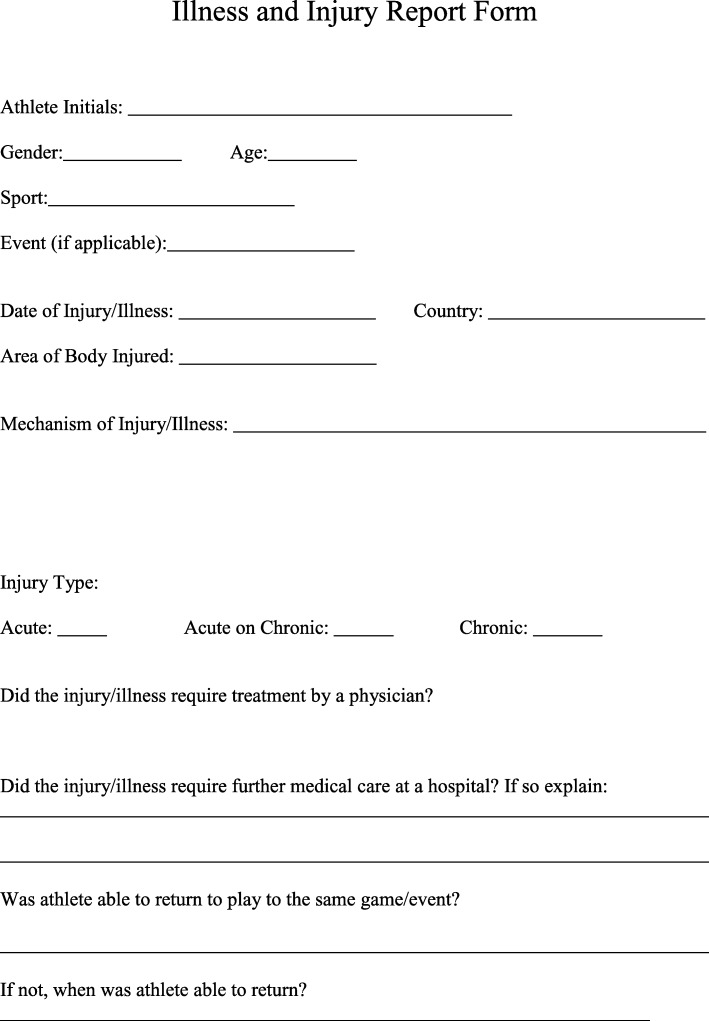
Injury and illness report form

During the Games, athlete interactions with either the physician or athletic training staff were documented with an “injury and illness report form” (Fig. 2). Variables recorded for each interaction included athlete initials, gender, age, country of origin, sport and event in which the injury occurred, date of injury/illness, area of body injured, mechanism of injury/illness, injury type, physician treatment, hospital referral, and when the athlete was able to return to play.

An injury or illness was described as any symptom that an athlete was experiencing that led them to seek medical consultation from either an athletic trainer or a physician present at the Games. An injury was further classified into one of three types: acute, chronic, or acute on chronic. An acute injury was defined as a new injury that occurred during the Games triggered by a precipitating event. A chronic injury was considered a previously existing injury without an acute triggering event during the Games. An acute on chronic injury was considered a previously existing injury that was exacerbated by an acute precipitating event during the Games.

## Results

409 athletes participated in the 2013 World Dwarf Games: 133 female and 276 male participants. A total of 29 athletes presented to medical staff for evaluation and treatment of injuries and illnesses. Of those, 4 injuries involved athletes but occurred outside of competition. Only 1 illness was reported during the week of games. A total of 24 competition related injuries were recorded among the 409 athletes (Table 3). These 24 injuries were the focus of this study with statistics excluding the 4 injuries that occurred outside of competition.

**Table 3:** Athlete injuries during the 2013 World Dwarf Games categorized by gender of athlete, acuity of injury, type of injury, and severity of injury along with epidemiologic incidence proportions

	Athletes Injured	% of Total Athletes Injured	Athletes at Risk	Incidence Proportion (%)
Total	24	100.0	409	5.9
Gender				
Male	20	83.3	276	7.2
Female	4	16.7	133	3.0
Acuity of Injury				
Acute	23	95.8	409	5.6
Acute-on-chronic	1	4.2	409	0.2
Chronic	0	0.0	409	0.0
Type of Injury				
Contusion	6	25.0	409	1.5
Sprain	3	12.5	409	0.7
Strain	10	41.7	409	2.4
Other^a^	5	20.8	409	1.2
I-RTP^b^	21	87.5		

^a^Other injuries include 3 hamstring cramps, 1 forehead laceration, and 1 IT band tendonitis

^b^“I-RTP” stands for immediate return to play, which means the athlete was given medical clearance to resume participation due to low severity of the injury. This was not documented for one athlete. Two other athletes reported their injuries towards the completion of the event, but could have returned if the competition had continued

The overall injury incidence rate, calculated as injuries per 100 athlete-competitions, was 0.78 (24 injuries in 3092 athlete competitions). The epidemiologic incidence proportion (IP) for male athletes was 7.2% and for female athletes was 3.0% (Table 3). In regards to acuity of injury, acute injuries were predominant (5.6% IP; Table 3). The most common type of injury was a muscle/tendon strain (41.7% of all injuries, 2.4% IP) followed by a bone/muscle contusion (25% of all injuries, 1.5% IP; Table 3). The sport with the highest injury incidence rate was soccer with 4.63 injuries per 100 athlete-competitions followed by basketball with 3.47 injuries per 100 athlete-competitions (Table 4). Lower extremity injuries were the most common in both soccer and basketball (100 and 71.4% of all injuries, respectively, Table 4). In regards to severity of injury, 87.5% of injured athletes immediately returned to play (I-RTP) after evaluation by medical staff (Table 4). Of the remaining injured athletes, I-RTP was not recorded for one and the other two could have returned if the competition had continued (injuries occurred near the end of their events). The only reported illness during the entire 2013 World Dwarf Games resulted in an illness incidence proportion of 0.2% (1 out of 409 athletes).

**Table 4:** Injury incidence rates for the sports with at least one injury recorded as well as categorization of injuries based on anatomical location

	Track and Field	Soccer	Basketball	Badminton	Floor Hockey
Injuries	1	12	7	2	2
Athlete-Competitions	842	259	202	327	183
Injuries per 100 Athlete-Competitions	0.12	4.63	3.47	0.61	1.09
Upper Extremity Injuries^a^	0	0	1	1	1
% of Total Injuries	0.0	0.0	14.3	50.0	50.0
Lower Extremity Injuries^b^	1	12	5	1	0
% of Total Injuries	100.0	100.0	71.4	50.0	0.0
Other Injuries^c^	0	0	1	0	1
% of Total Injuries	0.0	0.0	14.3	0.0	50.0

^a^Upper extremity injuries – 2 hand contusions, 1 finger sprain

^b^Lower extremity injuries – 3 hamstring cramps, 4 quadriceps strains, 3 knee strains, 2 ankle sprains, 2 hamstring strains, 1 quadriceps contusion, 1 knee contusion, 1 shin contusion, 1 ft contusion, 1 IT band tendonitis

^c^Other injuries – 1 forehead laceration, 1 low back strain

Among the different classification groups, the Open division had the highest incidence rate with 10.71 injuries per 100 athlete-competitions while the Futures division had the lowest with 0.00 injuries per 100 athlete-competitions (Table 5).

**Table 5:** Injury rate data based on age-defined competitions at the 2013 World Dwarf Games

	Injuries	Total Athlete-Competitions	Injuries per 100 Athlete-Competitions
Futures (ages 0–6)	0	63	0.00
Junior A (ages 7–11)**	1	472	2.12
Junior B (ages 12–15)**	1	594	1.68
Open (open to all ages)	18	1680	10.71
Masters (ages 35 and older)	1	283	3.53

*3 data points were excluded, as age was not recorded during the medical encounter

**There was an additional age division “Juniors – ages 10–12” for swimming as well as track & field events. Participants from these events were added to Junior A if ages 10–11 or Junior B if age 12

Injuries that occurred during the 2013 World Dwarf Games occurred in five sports: badminton, basketball, floor hockey, soccer, and track & field. Those injuries and their sports, including a brief review of the rule adaptations of the athletes, are included below.

### Badminton

Junior A, Junior B, Open, and Masters divisions participated in badminton during the 2013 World Dwarf Games. Separate singles competitions were conducted for males and females. Rule adaptions included junior-size badminton racquets being allowed for competitors in the Junior A and Junior B divisions. A sidearm serve was allowed because short stature and a long racket make the underhand serve difficult for the dwarf athlete. All other rules were consistent with the Parabadminton World Federation rules.

There were a total of 2 injuries during competition. One was a hamstring cramp and the other was a hand contusion that resulted from punching a pole. None of the injuries prevented an athlete from returning to play, either in the same game or in subsequent games.

### Basketball

Junior A, Junior B, and Open divisions participated in basketball during the 2013 World Dwarf Games. All teams were mixed-gendered. Rule adaptations included a regulation-sized court with 10 ft. high rims in the Open and Junior B divisions. An 8 ft. high rim was used for the Junior A division. An international size 6 (women’s size) basketball was used in the Open division while a size 5 (junior size) basketball was used for the Junior A and B divisions. Open division games consisted of four 8-min quarters. Junior division games consisted of four 6-min quarters. The remainder of the rules were in compliance with the International Basketball Federation (FIBA).

There were a total of 7 injuries during competition and one illness among basketball participants. The illness was an acute on chronic otitis media and was the only reported illness throughout the 2013 World Dwarf Games. Five of the seven reported basketball injuries were lower body acute injuries. One injury, an iliotibial band tendonitis, was classified as an acute on chronic injury. Other lower body injuries included 1 knee strain, 1 hamstring strain, 1 quadriceps strain, and 1 shin contusion. Upper body injuries included 1 forehead laceration resulting from a head to head collision and 1 index finger sprain. None of the injuries prevented an athlete from returning to play, either in the same game or in subsequent games.

### Soccer

Junior A, Junior B, and Open divisions participated in soccer during the 2013 World Dwarf Games, with separate men and women leagues. Each team consisted of 8 members (7 field players and 1 goalkeeper) on a 60 yd. long by 45 yd. wide field. The goal was 6 ft. high and 18 ft. wide. International size 4 balls were used for both Junior and Open divisions. Games consisted of two 25-min halves. Neither slide tackling nor headers were allowed. The remainder of the rules were in compliance with the Internationale de Football Association (FIFA).

There were a total of 12 injuries during the soccer games: 3 females and 9 males. This was the highest number of injuries for a particular event during the 2013 World Dwarf Games. All injuries were classified as acute and involved the lower extremities. Lower extremity injuries included 1 hamstring cramp, 3 quadriceps strains, 2 knee strains, 2 ankle sprains, 1 hamstring strain, 1 quadriceps contusion, 1 knee contusion, and 1 ft contusion. None of the injuries precluded the athlete from returning to play, either in the same game or in subsequent games.

### Floor hockey

Junior A, Junior B, Open, and Futures divisions participated in floor hockey during the 2013 World Dwarf Games in mixed gender teams. Each game consisted of three 10-min periods on a 35 × 70 ft. court surface. Each team consisted of 6 members: five field players and one goalkeeper. Pushing, tripping, hooking, blocking an opponent with the body, deliberately holding, or contacting another player were not allowed. The rules for the Futures division had a few different adaptations: three 6-min periods and three member teams with no goalkeepers.

A total of 2 injuries were recorded during Floor Hockey. One was a lower back strain and the other was a hand contusion. Both injuries were classified as acute. One athlete returned to play in the same game. The other athlete could have returned to play, but their injury occurred in the final moments of the competition and the game concluded before medical evaluation was completed.

### Track & Field

All divisions participated in Track events with separate competitions held for males and females. A wheelchair slalom event was held for athletes who used a manual or power wheelchair for most activities of daily living. Master and Open divisions Track events included the 100 m dash, 200 m dash, 4 × 100 m relay and the wheelchair slalom. Shorter runs (15 m, 20 m, 40 m and 60 m) were used for the Junior and Future divisions. International Paralympic Committee (IPC) rules were used for the Open division.

All divisions participated in Field events with separate competitions held for males and females. Field events included traditional discus, javelin, and shot put. Event modifications, including lighter weight equipment, were used for the Futures and Junior A divisions. International Paralympic Committee (IPC) rules were used for the Open division.

A total of 1 competition-related injury was recorded during the track & field portion of the 2013 World Dwarf Games. The injury was a hamstring strain; the athlete was able to run in their next event. Of note, there were 4 additional injuries that occurred outside of competition and were not included in calculation of injury rates and epidemiologic incidence proportions. One athlete suffered a head contusion and another had a shoulder strain following falls in the bleachers. The other two athletes presented with hand and wrist pain due to bee stings.

## Discussion

The main goal of this study was to document the incidence of injury and illness among participants in the 2013 World Dwarf Games. Based on the data collected it does appear that SD athletes can safely participate in the events offered during the World Dwarf Games. None of the 24 competition-related injuries or illnesses precluded the athletes from returning to play.

The second goal of this study was to identify any injury or illness patterns occurring during the Games. To our knowledge, this is the first study to investigate the injury and illness rates among SD athletes participating in athletic events. Understanding injury and illness patterns among this population subset is important for modifying rules necessary for injury prevention, ensuring participant safety, and allowing administrators and the organizing committee to plan for medical services at future events. With the overall injury incidence rate and epidemiologic incidence proportion during the 2013 World Dwarf Games being so low and no occurrence of serious injury that precluded a return to play, there seems to be little risk for participation in athletics within the dwarf population. One potential modification for athletic events at future World Dwarf Games could be having designated spectating areas for athletes. Two of the non-competition injuries during the track &field events were caused by falls from the bleachers and having a designated area for athletes with a safer platform could prevent future injuries. Considering most injuries occurred in contact sports, soccer and basketball, modifying rules to prevent excessive contact or adding larger penalties as a deterrent for such conduct may further reduce the incidence of injuries.

Overall epidemiologic incidence proportion documented at the 2013 World Dwarf Games was significantly lower than those previously documented in the Paralympic Games (Webborn et al., 2012; Ferrara et al., 2000). The number of participants in the World Dwarf Games is significantly lower than either the Summer or Winter Paralympic Games. Additionally, the Paralympic Games, especially the Winter Games, include events with high risk, such as alpine skiing and sledge hockey.

It is important to mention that many of the athletic events in the 2013 World Dwarf Games were adapted to accommodate athletes with SD. Contact sports were kept to a minimum and violations were penalized in some sports where contact is normally encouraged or expected, such as floor hockey. The only sports in which contact was allowed were basketball and soccer. As such, these sports presented with the highest injury incidence rates. Many of the sports accommodated the smaller stature of the athletes by incorporating smaller field sizes and smaller sized equipment, such as balls, goals, and rims.

Similar to previous reports (Willick et al., 2013; Webborn et al., 2006; Ferrara et al., 2000) acute injuries were more commonly reported than chronic injuries. Strains were the most commonly diagnosed acute injuries followed by contusions. Athletes with chronic injuries and illnesses may have been more likely to seek treatment from members of their own athletic staff or have been self-treating their ailments with a treatment plan developed by their medical staff prior to the Games.

The epidemiologic incidence proportion (IP) among male athletes was higher than that among female participants. This is unlike previous studies (Willick et al., 2013) that showed similar injury and illness IP between the sexes. One possible reason for a higher male IP in the 2013 World Dwarf Games was that more males than females participated in the two contact sports. The basketball division consisted of mixed gender teams while the only other contact sport, soccer, consisted of separate male and female divisions with a smaller female division.

When compared to other studies investigating illness and injury IP among Paralympic athletes, our study found an extremely low illness IP. Only one illness was reported during the entire 2013 World Dwarf Games. Other Paralympic studies (Derman et al., 2013; Ferrara et al., 2000) reported illness IP equal to or higher than the injury IP. This discrepancy in illness IP is unclear, but could be due to multiple unreported illnesses, definitions, and methodology rather than a true difference in illnesses. It may also be due to the smaller sample size of the 2013 World Dwarf Games when compared to sample sizes in the Paralympic Games. Additionally, the Paralympic Games consist of athletes with a wide variety of disabilities who may present with more medical complexities than in the dwarf population alone (Webborn & Van de Vliet, 2012).

Participation in athletics among athletes with disabilities should not be underestimated. Studies have shown that physical fitness can decrease cardiovascular risk in Paralympic athletes (Webborn & Van de Vliet, 2012). Additionally, participation in athletic events among athletes with SD can result in positive social consequences. Psychological stress and short-stature related stressors have been documented to be higher in children with achondroplasia (Nishimura & Hanaki, 2014). However, self-concept reporting from adult and youth athletes with disabilities has been shown to be similar to reporting from “able bodied” athletes (Sherrill et al., 1990). As a result, participation in athletics, including the World Dwarf Games, can positively impact social acceptance, behavior, and self-confidence among SD athletes, resulting in an improved quality of life.

There were several limitations to this study. Most studies that investigate injury and illness rates are prospective, but this study was retrospective. Advantages of prospective studies can include fewer potential sources of bias, confounding, and more accurate data collection than retrospective studies. Due to the retrospective nature of our study, we were unable to evaluate mechanisms of injuries due to insufficient details provided on the report forms. In addition, the ages of three athletes were not recorded, which would have an impact on the calculated injury incidence rates based on age. Multiple administrative barriers, e.g. limited resources and the inability to obtain informed consent from all athletes, including minors and non-English language participants, prohibited prospective IRB approval. Consequently, reporting of medical encounters was requested by the Dwarf Athletic Association of America in an effort to identify areas of possible quality improvement during the 2013 World Dwarf Games. With post-hoc IRB approval, the case series of medical encounters was retrospectively analyzed. Future prospective studies involving the World Dwarf Games are needed to ensure appropriate and accurate data collection of age, mechanism of injury, and severity of injury (grading sprains/strains in addition to immediate return to play decision).

Recent sports medicine literature has incorporated exposure data into injury and illness rate calculations. While we were able to determine the number of athletes participating in each event, accurate calculation of time-based exposure data (minutes or hours of competition) could not be performed. We recommend that future prospective studies are needed in which time-based exposure data is collected to provide a more accurate calculation of injury and illness rates.

Finally, it is possible that not all injuries and illnesses were reported. Since the illness IP in our study was so low compared to many previous studies, it is possible that illnesses were unreported, missed or treated at outside medical facilities, such as local medical offices or urgent care clinics. Advertising the availability of medical personnel and data collection goals during the registration period or the Opening Ceremonies of future World Dwarf Game competitions could be useful.

## Conclusions

Based on the data collected, it does appear that athletes with skeletal dysplasia can safely participate in the events offered during the World Dwarf Games. Muscle/tendon strains were the most commonly diagnosed acute injuries with the injury incidence rate among males being higher than female participants. With the injury and illness IP during the 2013 World Dwarf Games being so low, and no occurrence of serious injury that precluded a return to play, there seems to be little risk for participation in athletics within the dwarf population. Additional data collected in future events can identify trends, which may lead to improvements in preparation and safety in later events. Adding a designated spectator area for athletes as well as modifying rules to prevent excessive physical contact in soccer and basketball competitions may reduce the incidence of injury. In addition, the results of this study should encourage people with skeletal dysplasia to participate in athletics. Physical fitness has been shown to decrease cardiovascular risk, improve mental health and quality of life in this population.

## Abbreviations

IP: incidence proportion
I-RTP: immediate return to play
SD: skeletal dysplasia

## Authors’ information

Dr. Saffarian is a faculty member at Michigan State University Health Team. He is a graduate of the College of Osteopathic Medicine at Michigan State University. He completed his residency in Physical Medicine and Rehabilitation at MSU. He is fellowship-trained in Electromyography, Interventional Spine and Sports Medicine.
